# Two Complete Genome Sequences of *Squash mosaic virus* from 20-Year-Old Cucurbit Leaf Samples from Australia

**DOI:** 10.1128/genomeA.00778-17

**Published:** 2017-08-10

**Authors:** Solomon Maina, Owain R. Edwards, Roger A. C. Jones

**Affiliations:** a School of Agriculture and Environment, Faculty of Science, The University of Western Australia, Crawley, Western Australia, Australia; b Institute of Agriculture, Faculty of Science, The University of Western Australia, Crawley, Western Australia, Australia; c Cooperative Research Centre for Plant Biosecurity, Canberra, Australian Capital Territory, Australia; d CSIRO Land and Water, Floreat Park, Western Australia, Australia; e Department of Agriculture and Food Western Australia, South Perth, Western Australia, Australia

## Abstract

We present the first complete Australian *Squash mosaic virus* (SqMV) genome sequences. We compared the 2 Australian genomes from 20-year-old cucurbit samples with 8 other SqMV genomes. The Australian genomes shared >99.0% nucleotide identities, and their RNA1 and RNA2 sequences most closely resembled isolates Y and Kimbe from Japan, respectively.

## GENOME ANNOUNCEMENT

Possible genetic connectivity between viruses infecting crops in northern Australia and nearby southeast Asian countries is being investigated in a biosecurity project (1–11). *Squash mosaic virus* (SqMV) (genus *Comovirus*, family *Secoviridae*) (12, 13) was absent from this project’s cucurbit samples (8, 9) despite occurring in northwest Australia (14). SqMV, which causes a serious disease of cucurbits, is transmitted by beetles and plant-plant contact and is readily seedborne in cucurbits (12, 15). In 1996, leaf samples were collected from five symptomatic cucurbit crop plants in the Ord River Irrigation Area near Kununurra, East Kimberley, northwest Australia. When sap from sample 702K was examined by electron microscopy, it contained 30-nm-diameter icosahedral particles typical of SqMV (16). Leaf tissue from all five samples was preserved over silica gel for 20 years in a Queensland culture collection before being returned in 2016. Complete SqMV genomes were obtained from sample 702K from watermelon and sample 697K from honeydew melon; sample collection 697K also contained *Zucchini yellow mosaic virus* (genus *Potyvirus*, family *Potyvirideae*) (9). SqMV has a bipartite single-stranded RNA genome consisting of RNA1 and RNA2 (12, 13, 17–19).

Total RNA extracts were treated with RNase-free DNase (Invitrogen), and after quality control procedures the RNA was subjected to RNA-seq library preparation using a Ribo-Zero plant kit (catalogue no. RS-122-2401, Illumina) (1–11, 20, 21). Sequencing was conducted by Macrogen Inc. using HiSeq 2500 with a Truseq SBS kit (Illumina) with 151 cycles to generate paired-end reads in a multiplex of 24 samples per lane. Reads were assembled and genomes annotated using CLC Genomics Workbench 6.5 (CLC bio) and Geneious 8.1.7 (Biomatters) (22). Further alignment was performed by MAFFT (23).

Samples 702K and 697K yielded 18,860,882 (702K) and 19,260,418 (697K) reads and, after trimming, 18,645,226 (702K) and 19,014,605 (697K) reads. *De novo* assembly generated 186 contigs with 501,319 to 1,021,722 reads (702K) and 638 contigs with 4,356,590 to 5,860,309 reads (697K). These mapped to the contigs of interest with coverage of 140,122 to 180,469 (702K) and 19,144 to 24,594 (697K) times. Both genomes contained RNA1 and RNA2. The sequence lengths obtained with each were 5,821 (RNA1) and 3,333 (RNA2) nucleotides (nt), and blast searches revealed SqMV (24). The RNA2 genome codes for movement peptide, large capsid peptide, and small capsid peptide. The RNA1 codes for cofactor peptide, helicase peptide, VPg peptide, protease peptide, and RNA-dependent polymerase peptide (18). Such coding is typical for comoviruses (25, 26). The RNA1s and RNA2s from samples 697K and 702K shared 99.1% and 99.4% nt identities, respectively. Compared to the 8 genomes in GenBank, none of which were southeast Asian, their RNA1s most closely resembled those of Japanese isolate Y with 89.3% (702K1) and 89.5% (697K1) nt identities, and their RNA2s most closely resembled those of Japanese isolate Kimbe with 98.5% (702K2) and 98.4% (697K2). To establish whether any genetic connectivity exists, SqMV genomes from southeast Asian countries and more from Australia are needed.

### Accession number(s).

The genome sequences have been deposited in GenBank with the accession numbers MF166754 through MF166757.
